# Gene Regulatory Networks of *Penicillium echinulatum* 2HH and *Penicillium oxalicum* 114-2 Inferred by a Computational Biology Approach

**DOI:** 10.3389/fmicb.2020.588263

**Published:** 2020-10-27

**Authors:** Alexandre Rafael Lenz, Edgardo Galán-Vásquez, Eduardo Balbinot, Fernanda Pessi de Abreu, Nikael Souza de Oliveira, Letícia Osório da Rosa, Scheila de Avila e Silva, Marli Camassola, Aldo José Pinheiro Dillon, Ernesto Perez-Rueda

**Affiliations:** ^1^Unidad Académica Yucatán, Instituto de Investigaciones en Matemáticas Aplicadas y en Sistemas, Universidad Nacional Autónoma de Mexico, Mérida, Mexico; ^2^Laboratório de Bioinformática e Biologia Computacional, Instituto de Biotecnologia, Universidade de Caxias do Sul, Caxias do Sul, Brazil; ^3^Departamento de Ciências Exatas e da Terra, Universidade do Estado da Bahia, Salvador, Brazil; ^4^Departamento de Ingeniería de Sistemas Computacionales y Automatización, Instituto de Investigaciones en Matemàticas Aplicadas y en Sistemas, Universidad Nacional Autónoma de Mexico, Ciudad Universitaria, Mexico; ^5^Laboratório de Enzimas e Biomassas, Instituto de Biotecnologia, Universidade de Caxias do Sul, Caxias do Sul, Brazil; ^6^Facultad de Ciencias, Centro de Genómica y Bioinformática, Universidad Mayor, Santiago, Chile

**Keywords:** *Penicillium*, regulatory network, orthologous, gene regulation, genomics, fungi

## Abstract

*Penicillium echinulatum* 2HH and *Penicillium oxalicum* 114-2 are well-known cellulase fungal producers. However, few studies addressing global mechanisms for gene regulation of these two important organisms are available so far. A recent finding that the 2HH wild-type is closely related to *P. oxalicum* leads to a combined study of these two species. Firstly, we provide a global gene regulatory network for *P. echinulatum* 2HH and *P. oxalicum* 114-2, based on TF-TG orthology relationships, considering three related species with well-known regulatory interactions combined with TFBSs prediction. The network was then analyzed in terms of topology, identifying TFs as hubs, and modules. Based on this approach, we explore numerous identified modules, such as the expression of cellulolytic and xylanolytic systems, where XlnR plays a key role in positive regulation of the xylanolytic system. It also regulates positively the cellulolytic system by acting indirectly through the cellodextrin induction system. This remarkable finding suggests that the XlnR-dependent cellulolytic and xylanolytic regulatory systems are probably conserved in both *P. echinulatum* and *P. oxalicum*. Finally, we explore the functional congruency on the genes clustered in terms of communities, where the genes related to cellular nitrogen, compound metabolic process and macromolecule metabolic process were the most abundant. Therefore, our approach allows us to confer a degree of accuracy regarding the existence of each inferred interaction.

## 1. Introduction

The connectivity between biological data facilitates the inference of networks. These graphs are made up of nodes and connections between them, comprising a flexible model that can capture the complexity and interconnectivity of biological information (Huber et al., 2007). The elucidation of regulatory relationships between transcription regulators and their target genes is essential to understand various biological processes. These processes range from cell growth and division, cell differentiation in multicellular organisms and cell response to environmental changes. In addition, networks are often identified as the layer that connects genomic data to phenotypic characteristics (Carter et al., 2013).

A gene regulatory network (GRN) is a directed graph in which gene regulators are connected to target genes (TGs) by interaction edges (Karlebach and Shamir, 2008). In addition to transcription factors (TFs) that can act as both activators and repressors, gene regulators also include RNA-binding proteins and regulatory RNAs. This type of network addresses a key challenge in experimental and computational biology, helping to clarify the relationships between genes and the products they encode (Jackson et al., 2020). GRNs combined with knowledge mining has a major potential to improve omics data interpretation, allowing the discovery of how transcription regulation may control biological processes, phenotypes, and diseases (Hassani-Pak and Rawlings, 2017).

To date, GRNs have been reconstructed only for a few model organisms (Gerstein et al., 2012; Chen et al., 2018; Hu et al., 2018; Jackson et al., 2020), since their reconstruction depends largely on experimental approaches. On the other hand, GRN inferences become a viable alternative (Filho et al., 2019; Staunton et al., 2019) supported by several information resources and bioinformatic tools (Glenwinkel et al., 2014; Penfold et al., 2015; Lam et al., 2016; Koch et al., 2017; Kulkarni et al., 2017).

GRN inference resources may be categorized into six classes, according to the approach employed and the underlying data used: Coexpression, Sequence Motifs, Chromatin Immunoprecipitation (ChIP), Orthology, Literature, and Protein-Protein Interaction (PPI) specifically focused on transcriptional complexes. The more information is aggregated, the more accurate a TF-TG relationship becomes. In particular, GRNs can benefit from orthology-based knowledge from closely related species; where the key concept is that a TF-TG relationship proven in one organism can be conserved in another one (Mercatelli et al., 2020). However, this knowledge transfer requires reliable methods to define orthology between different genes for any TF-TG pair, as well as taking into account the phylogenetic positioning of the species analyzed (Fernandez-Valverde et al., 2018).

Orthologous genes are the most similar genetic elements in different species, in terms of sequence, structure, and function (Gabaldón and Koonin, 2013). Detecting orthology is especially important to maximize information content and accuracy. Therefore, the premise of constructing a TF-TG network is based on the presence of genes that can be traced to a common ancestor between different species, also taking into account functionality besides nucleotide sequence (Mercatelli et al., 2020).

Another widespread GRN-inference resource comprehends sequence motifs. This resource comprises the identification of conserved DNA sequence motifs recognized by TFs in the regulatory region of genes. These motifs, known as transcription factor binding sites (TFBSs), can be represented in the form of a position frequency matrix (PFM) or a position weight matrix (PWM) (Hu et al., 2013), which may be useful to increase the accuracy of TF-TG relationships (Mercatelli et al., 2020). Recent studies, characterizing the specificity of TFs in eukaryotes, include the representation of several TFBSs in the form of matrices for model microorganisms such as *Aspergillus nidulans, Neurospora crassa*, and *Saccharomyces cerevisiae* (Lambert et al., 2019).

The identification of TFBSs is based on the assumption that some non-coding regions among related species are likely to be under negative selection and therefore contain conserved functional motifs (Hu et al., 2013). Cis-regulatory elements can be conserved in more distantly related species, even when the orthologous regulatory regions are divergent to be precisely aligned. However, despite the conservation of these motifs, the regulation role may not be conserved, suggesting possible different functions (Gasch et al., 2004).

So far, in the fungi scope, *S. cerevisiae* S288C, *N. crassa* OR74A, and *A. nidulans* FGSC A4 have in-depth studies for GRN reconstruction (Hu et al., 2018; Jackson et al., 2020), whereas, for the genus *Penicillium*, no global GRNs have been described. The reconstruction of GRN in *S. cerevisiae* was, in particular, facilitated by the YEASTRACT+ database, which gathers interaction information for this organism, comprising 12,228 interactions (Jackson et al., 2020; Monteiro et al., 2020). In this regard, curated data of *A. nidulans* and *N. crassa* regulatory interactions may be useful as a catalog for gene regulation studies in filamentous fungi, since 33 conserved regulatory interactions, supported by classical experiments were identified in both species (Hu et al., 2018).

The 2HH wild-type was previously classified by morphology as *Penicillium echinulatum* in the 80's. Long-term 2HH strain improvement studies use this classification (Camassola et al., 2004; Dillon et al., 2006, 2011; Camassola and Dillon, 2007a,b, 2009, 2010, 2012; Rubini et al., 2010; Ribeiro et al., 2012; Dos Reis et al., 2013; Novello et al., 2014; Schneider et al., 2014, 2016, 2018, 2020). However, whole genome sequences of 2HH strain, deposited recently at GenBank and released in this work, provided evidence that 2HH strain is closely related to *P. oxalicum*, suggesting a taxonomic study for the repositioning and characterization of this strain. The close relationship between these two filamentous fungi leads to a combined study, both *P. echinulatum* 2HH and *P. oxalicum* 114-2 (Liu et al., 2013d) are well-known cellulase producers studied extensively in Brazil and China, respectively. Despite advances to understand the mechanisms responsible for regulating the expression of cellulases in *P. oxalicum* (Liu et al., 2013a,b,c,d; Li et al., 2015; Yao et al., 2015), studies addressing the global mechanisms of gene regulation of these two important organisms of biotechnological interest are scarce. It also highlights the relevance of *Penicillium* combined studies due to its potential superiority over existing cellulase producers (Vaishnav et al., 2018).

In the present work, we propose the inference of GRNs for *P. echinulatum* 2HH and *P. oxalicum* 114-2, based on TF-TG orthology relationships of three related species with well-known regulatory interactions, combined with TFBSs prediction. First, GRNs of related species (*A. nidulans, N. crassa*, and *S. cerevisiae*) allow the mapping of orthologous interactions. Further, the TFBSs prediction provides accuracy to TF-TG relationships. The reconstructed GRNs were posteriorly analyzed in terms of topology, identifying TFs as hubs, and modules were inferred by using a community approach algorithm. Therefore, our approach allows us to confer a degree of accuracy regarding the existence of each inferred interaction.

## 2. Materials and Methods

**Figure 1:**
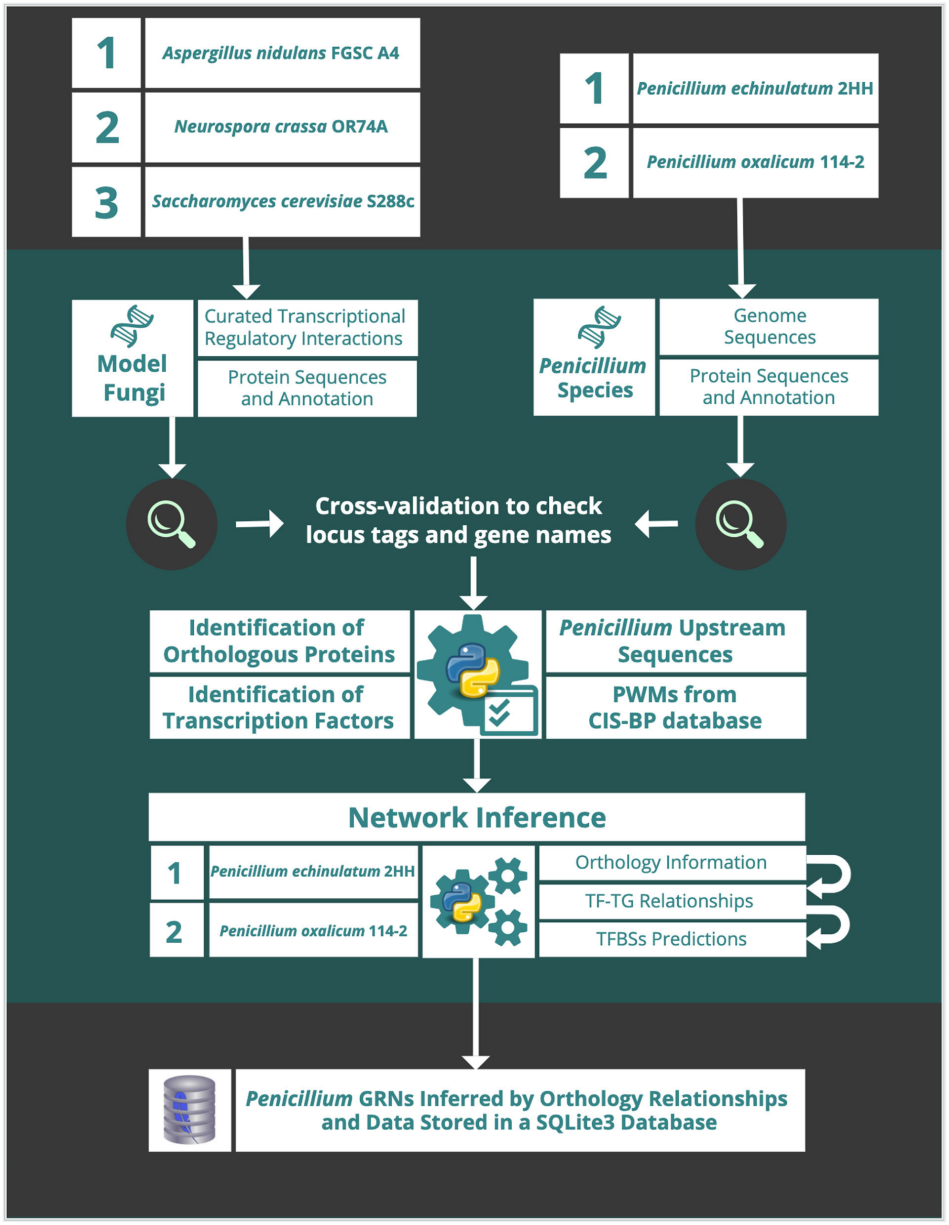
Schematic workflow of the network inference procedure steps.

The schematic workflow (Figure 1) describes procedure steps of the network inference. Details on each step herewith the input and output data are described below.

### 2.1. Fungal Genomes Analyzed

The information of five fungal genomes and proteomes used in this study were downloaded from the NCBI server for (a) *P. echinulatum* 2HH (GCA_014839855.1 UCS_PECH_1.0); (b) *P. oxalicum* 114-2 (GCA_000346795.1 pdev1.0); (c) *A. nidulans* FGSC A4 (GCF_000149205.2 ASM14920v2); (d) *N. crassa* OR74A (GCF_000182925.2 NC12); and (e) *S. cerevisiae* S288c (GCF_000146045.2 R64).

### 2.2. Identification of Orthologous Proteins

To identify orthologous proteins between each *Penicillium* proteome and the proteomes of *A. nidulans, N. crassa*, and *S. cerevisiae*, we used the program ProteinOrtho (V6.0.15) (Lechner et al., 2011). ProteinOrtho analyses were performed with default parameters, except by the report of singleton genes without any hit. Further, OrthoVenn2 (Xu et al., 2019) was used to identify orthologous clusters in the five proteomes and to perform GO enrichment for each cluster.

### 2.3. Identification of Transcription Factors

To assess TFs diversity, protein sequences of whole proteomes were used to search TF domains using InterProScan (v5.25-64.0) (Jones et al., 2014) and hmmscan (v3.1b2) (Potter et al., 2018). InterProScan was used to map Interpro families and domains, while hmmscan was used to identify domains over the PFAM database (v31.0-2017-02) (El-Gebali et al., 2019) using default parameters. Afterwards, PFAM and InterPro predictions of each species were compiled making use of the 91 DNA-binding domains described in the catalog of the main eukaryotic transcription factor families (Weirauch and Hughes, 2011), also used by CIS-BP database (Weirauch et al., 2014). TF distribution is available in Supplementary Table 1. Finally, a heatmap was generated including 37 of the 91 domains, which were found in the analyzed fungal proteomes (Supplementary Material **Script “funregulation_tf_heatmap.py”**).

### 2.4. Collection of Regulatory Interactions From Related Species

Regulatory interactions from *A. nidulans, N. crassa* (Hu et al., 2018), and *S. cerevisiae* available in YEASTRACT+ (Monteiro et al., 2020) were collected and organized in tab-delimited files. All interactions are available in Supplementary Table 2. A cross-validation was performed to check locus tag and gene name for each regulatory interaction, crossing information from the reference genomes and regulatory interactions. This step was essential to guarantee input data accuracy, especially for *S. cerevisiae* regulatory interactions, as it had already been described in other fungal models (Hu et al., 2018). It is important to note that some locus tags and/or gene names were corrected and others were not found in the *S. cerevisiae* reference genomes (SGD and Genbank), and therefore have been discarded from this study.

### 2.5. Upstream Sequences

Annotation in gff3 format and whole genome sequences of *P. echinulatum* 2HH and of *P. oxalicum* 114-2 were used to extract the DNA sequences comprising 1000bp upstream of each gene (Supplementary Material **Script “funregulation_promoter_extract.py”**).

### 2.6. Weight Matrices Used to Identify TFBS

PWMs from *A. nidulans, N. crassa*, and *S. cerevisiae* were obtained from CIS-BP Database (Weirauch et al., 2014). A cross-validation was also performed to check locus tag and gene name for each transcription factor, crossing information from the reference genomes and CIS-BP. Some locus tags and/or gene names were corrected, especially for *A. nidulans*.

### 2.7. SQLite3 Database

The large volume of data and its interconnectivity restricts the access to specific records in text files and makes it impossible to load complete files on machines with low memory capacity. Consequently, we chose a SQLite database by modeling six tables: “gene,” “ortho,” “pwm,” “regulation,” “tfbs_prediction,” and “network_node.” Input data obtained in the previous steps were inserted in the tables: “gene,” “ortho,” “pwm,” and “regulation” (see Supplementary Material).

### 2.8. Inference of Global Regulatory Networks (GRN)

In order to reconstruct the GRN of *P. echinulatum* 2HH and *P. oxalicum* 114-2, a number of steps were considered, as described below (Figure 1).

#### 2.8.1. Identification of TF-TG Relationships by Orthology

The identification of potential TF-TG interactions in *P. echinulatum* 2HH and in *P. oxalicum* 114-2 was performed (Supplementary Material **Script “funregulation_network_inference.py”**). This step considered the regulatory interactions from *A. nidulans, N. crassa*, and *S. cerevisiae* in addition to the orthology relationships previously mapped. For each known TF-TG interaction of these three species, only when orthologous were found for both TF and TG in the *Penicillium* species, a new TF-TG interaction was created for the respective *Penicillium* species and these data were inserted in the “regulation” table. Foreign-keys cross reference from which species derived each new TF-TG interaction of the *Penicillium* species. This information allows us to analyze the conservation of TF-TG interactions between the species analyzed. Besides, it also allows us to assign different weights for the new TF-TG interactions, according to the species phylogenetic distances of which the TFs-TGs interactions were inferred.

#### 2.8.2. TFBSs Prediction

For each TF-TG interaction, TFBS prediction was carried out. RSAT matrix-scan (Turatsinze et al., 2008) was used to predict the TFBSs using all the respective PWMs from related species, obtained from CIS-BP Database (Weirauch et al., 2014). RSAT matrix-scan analyses were performed with “cis-bp” as matrix format. Other default parameters were maintained, including an *e*-value <1e-4 as upper threshold *P*-value. RSAT results for each TF-TG interaction were stored in single text files and also in the “tfbs_prediction” table.

#### 2.8.3. Network Nodes

For *P. echinulatum* 2HH and in *P. oxalicum* 114-2, unique TF-TG interactions were inserted in the “network_node” table, checking out from which model organisms the relationship originated and counting how many TFBS were predicted for it (**Supplementary Material Script “funregulation_network_inference.py”**). All data from the “network_node” and “tfbs_prediction” tables were exported to the tab-delimited output files.

#### 2.8.4. Supplementary Material

Finally, the GRN inference was performed by Python scripts (Python Software Foundation, 2020) (v3.8.2) and data were stored in a SQLite database (SQLite Consortium, 2020) (v.3.31.1). All scripts and data are available in the FunRegulation project at Git http://www.github.com/alexandrelenz/funregulation.git.

### 2.9. Topological Analysis of the Networks

In order to topologically characterize the GRN, numerous metrics, such as node degree, clustering coefficient, centrality, hubs, and communities were determined (Junker and Schreiber, 2011). Degree of a node (*K*) is defined as the number of interactions that it has with other nodes. In directed networks, input (*Kin*) and output degree (*Kout*) are defined as the number of arrows that enter and leave from a node, respectively, which corresponds to the number of TFs that affect a certain TG, and the number of TGs that a TF regulates (Barabási and Oltvai, 2004).

Centrality (*C*) is a function which assigns every *v* ∈ *V* of a given graph *G*, where the value *C*(*v*) ∈ ℜ. Thus, to get a ranking of the node for a given *G* we choose the convention that a node *u* is more important than a node *v* if *C*(*u*) > *C*(*v*). Lastly, we computed assorted centrality metrics, including degree, closeness, betweenness, and eigenvector centrality (Junker and Schreiber, 2011).

In a network, connectivity refers to the connections between each pair of nodes, and these connections can be via a direct or indirect link. Therefore, the connected component was defined as a set of nodes that are linked to each other by paths and give us information about how much the elements are connected in a network and their module structure (Junker and Schreiber, 2011).

To identify communities, we used the algorithm proposed by Blondel et al. (2008), that assigns a different community to each node of the network. When a node is moved to one of its neighbors' community, it achieves the highest positive contribution to modularity. This step is repeated for all nodes until no further improvement can be reached. Then, each community is considered as a single node on its own, and a subsequent move is repeated until there is only a single node left or when the modularity cannot be increased in a single step.

## 3. Results and Discussion

### 3.1. Identification of Common Proteins in All the Genomes

**Figure 2:**
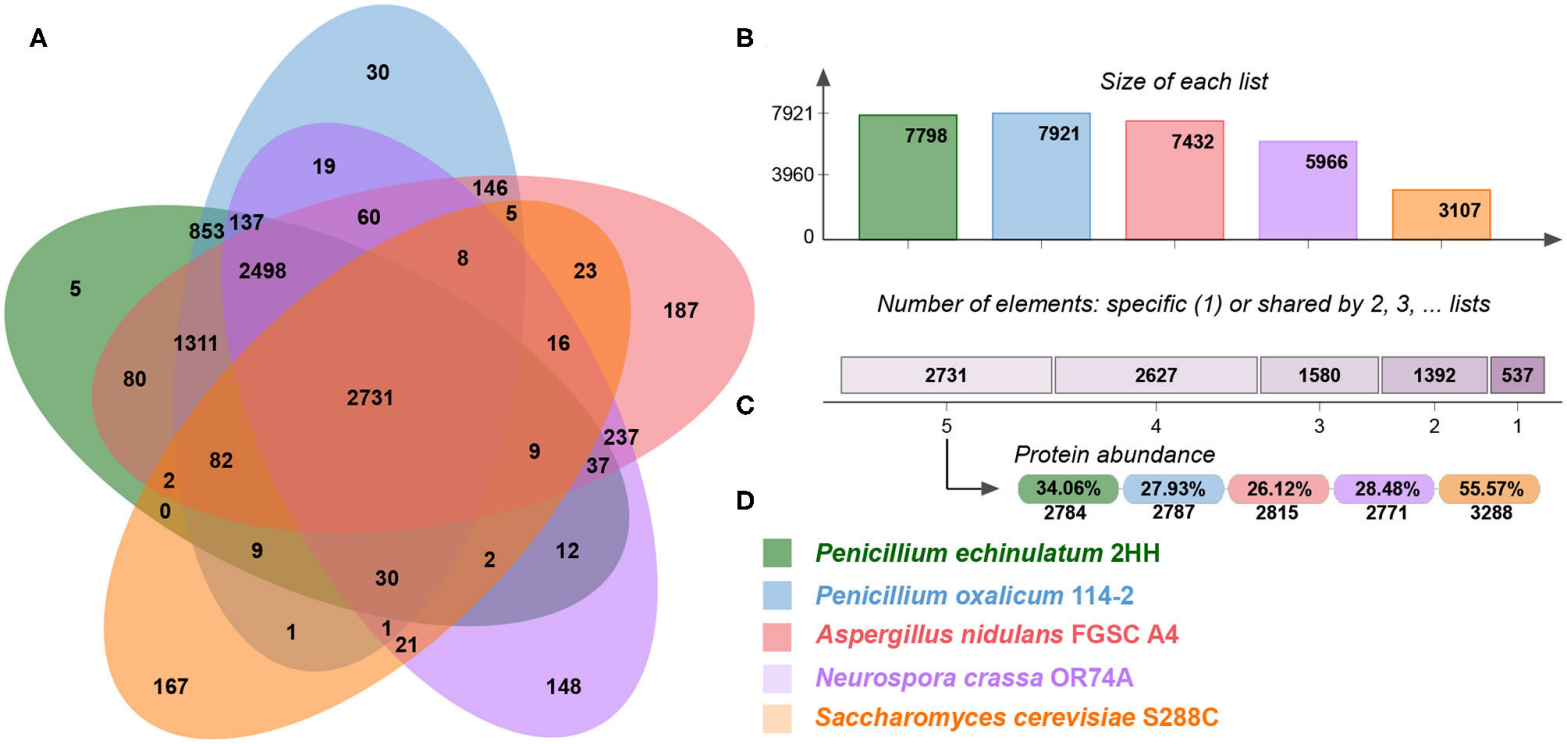
**(A)** Venn diagram comparing orthologous clusters of whole-proteomes. **(B)** The barplot shows the number of orthologous clusters by organism. **(C)** The plot indicates the number of clusters that are organism-specific or shared by 2, 3, 4, or 5 organisms. **(D)** For the 2,731 clusters shared by 5 organisms, the protein abundance in percentage and absolute numbers are shown for each organism.

One of the premises for knowledge transfer of regulatory relationships is a close phylogenetic positioning of the selected species, resulting in a high rate of shared orthologous genes. In this study, we use regulatory relationships of three model fungi (*A. nidulans, N. crassa*, and *S. cerevisiae*) for regulatory transfer to *P. echinulatum* and *P. oxalicum*. In order to analyze shared orthologous proteins, the complete proteomes were displayed into OrthoVenn2 (Figure 2). The Veen diagram indicates that 2,731 clusters of protein orthologous were identified as common to all proteomes, corresponding to 36.06 and 27.93% of *P. echinulatum* and *P. oxalicum* proteomes, respectively. The diagram also displays 5 clusters including 11 proteins exclusively associated to *P. echinulatum* and 30 clusters containing 70 proteins exclusively associated to *P. oxalicum*, suggesting that those proteins are species-specific.

A functional analysis of the 14,445 proteins placed in the 2,731 clusters identified as common to all proteomes, showed that DNA-dependent transcription (GO:0006351) (*p*-val: 6.13e-21) and rRNA processing (GO:0006364) (*p*-val: 1.37e-14) are the most represented GO terms. This functional analysis denotes that the core proteins shared between the five fungal species includes proteins related to cellular synthesis of RNA on a template of DNA (transcription regulator activity) and conversion of rRNA transcripts into mature rRNA molecules (rRNA maturation). These results suggest that probably the vast majority of transcription factors are conserved in the five fungal species, supporting the transference of regulatory knowledge from the model fungi to the *Penicillium* species.

### 3.2. The Repertoire of TFs Comprises 37 Families

The TFs (TFome) repertoire of a species comprises a set of essential proteins responsible for the regulation of gene expression in a cell. Around 80 families of TFs have been described in fungi and the proportion of transcription factors in genomes increases as a function of genome size, where larger genomes have more TFs. However, the increment is largely limited to three main families, Zn2Cys6 clusters, C2H2-like Zn fingers, and homeodomain-like (Shelest, 2017).

**Figure 3:**
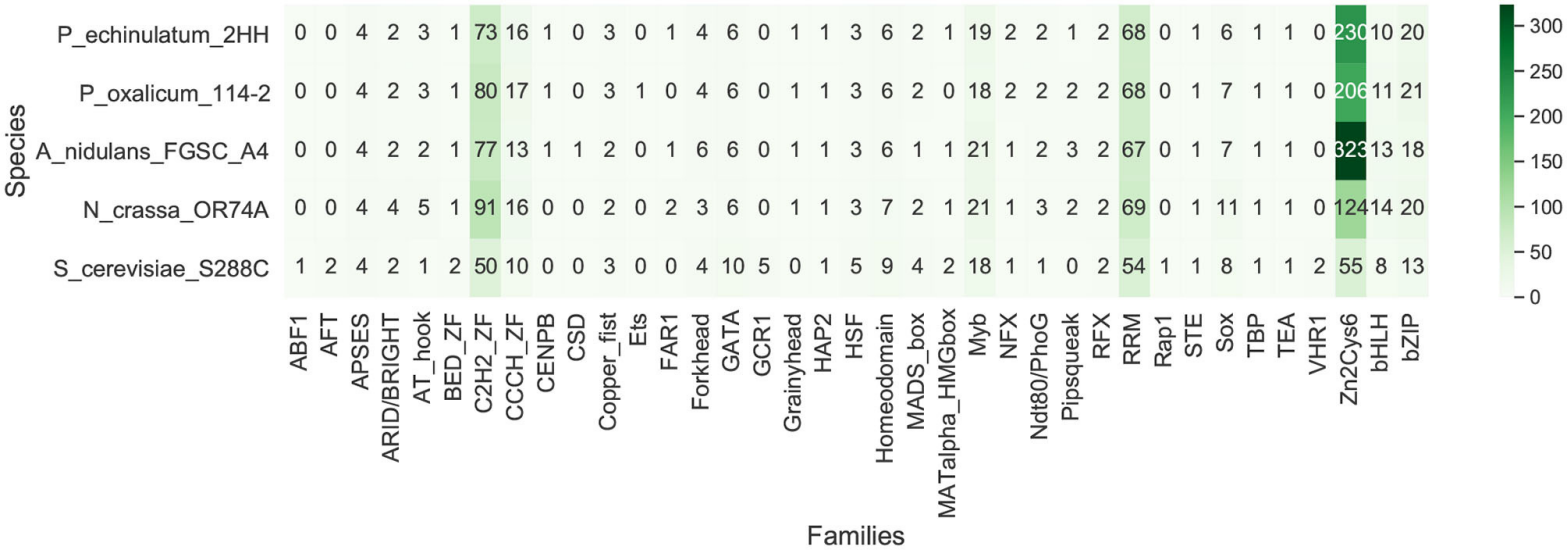
Abundance and distribution of transcription factor families in fungal genomes. TFs of *P. echinulatum* 2HH containing multiple domains: Zn2Cys6+C2H2_ZF (5); Homeodomain+C2H2_ZF (1); BED_ZF+C2H2_ZF (1); STE+C2H2_ZF (1); CCCH_ZF+NFX (1); RRM+CCCH_ZF (3); RRM+Zn2Cys6 (1). TFs of *P. oxalicum* 114-2 containing multiple domains: Zn2Cys6+C2H2_ZF (7); Homeodomain+C2H2_ZF (1); BED_ZF+C2H2_ZF (1); STE+C2H2_ZF (1); CCCH_ZF+NFX (1); RRM+CCCH_ZF (3).

We compiled InterPro and PFAM predictions in each fungal species, taking as reference the catalog of 91 DNA-binding domains of the main eukaryotic transcription factor families (Weirauch and Hughes, 2011), also used by CIS-BP database (Weirauch et al., 2014). We found 37 TF families of the catalog in the analyzed fungal proteomes, depicting the diversity of TF families shown in Figure 3. These families include the most important TFs of the five species, previously described in the literature. The identified TFome of *P. echinulatum* and *P. oxalicum* include 478 and 463 TFs, respectively.

From this analysis, we found that the largest family of TFs in *P. oxalicum* and *P. uscensis* corresponds to the fungal-specific Zn2Cys6 binuclear cluster. Zn2Cys6 binuclear clusters have been identified in all fungal species analyzed so far. For example, Zn2Cys6 zinc finger proteins, such as AmyR (PDE_03964), XlnR (PDE_07674) (Li et al., 2015), and AraR (PDE_04461) (Gao et al., 2019) act in the regulation of carbon metabolism in *P. oxalicum* 114-2. Based on this knowledge, this domain could be considered ubiquitous to fungi. Based on its abundance and distribution, it has been proposed that Zn2Cys6 clusters expand much faster in ascomycetes when correlated to proteome size growth, suggesting a particular role in the evolutionary history of this phylum (Shelest, 2017). In this regard, Zn2Cys6 clusters have been found in proteins regulating a wide range of processes, including carbon and nitrogen metabolism, amino acid and vitamin synthesis, stress response, pleiotropic drug resistance, meiosis, and morphogenesis, to name but a few (MacPherson et al., 2006).

The second more abundant family identified in both *Penicillium* corresponds to the highly conserved family of C2H2 zinc fingers. The functional roles associated with members of this family are extraordinarily diverse and include DNA recognition, transcription, mRNA trafficking, cytoskeleton organization, epithelial development, chromatin remodeling, and zinc sensing, amongst others (Laity et al., 2001). In *P. oxalicum* 114-2, a C2H2 transcription factor FlbC (PDE_08372) regulates fungal asexual development and acts as an essential activator of genes encoding cellulases, hemicellulases, and other proteins with functions in lignocellulose degradation (Yao et al., 2016). Another C2H2 transcription factor BrlA (PDE_00087) has not only a key role in regulating conidiation, but it also regulates secondary metabolism extensively as well as the expression of cellulase genes (Qin et al., 2013).

At the same time, two other relevant TF families in fungi are the basic leucine zipper (bZIP) and the zinc finger GATA-type. In fungi, bZIP comprehends important regulation mechanisms, responding to oxidative stress, DNA-damage, and amino acid starvation (Tian et al., 2011). The bZIP transcription factor ClrC (PDE_09023) in *P. oxalicum* 114-2, positively regulates multiple stress responses, conidiation and the transcription levels of major cellulase genes, as well as two cellulase transcriptional activator genes, ClrB and XlnR (Lei et al., 2016). Still in *P. oxalicum* 114-2, another bZIP transcription factor CpcA (PDE_08488), is a conserved transcriptional activator for the cross-pathway control of amino acid biosynthetic genes, supporting normal growth and extracellular enzyme production under amino acid non-starvation condition (Pan et al., 2020). GATA-type fungal TFs regulate nitrogen metabolism, light induction, siderophore biosynthesis, and mating-type switching, playing global roles in growth and development (Scazzocchio, 2000). Some TFs of this family are widely studied, for example, the light-responsive WC-1 and WC-2 in *N. crassa* (Grimaldi et al., 2006), or the nitrogen regulators AreA and AreB in *A. nidulans* (Macios et al., 2012). In *P. oxalicum* HP7-1, the GATA-type transcription factor NsdD, ortholog to PDE_02029 in *P. oxalicum* 114-2, regulates the expression of major genes involved in starch, cellulose, and hemicellulose degradation, conidiation, and pigment biosynthesis (He et al., 2018). In *A. nidulans*, NsdD is an activator of sexual development and key repressor of conidiation (Han et al., 2001).

A considerable number of TFs identified in low proportions were identified in both fungal genomes, as similar to the model organisms. As examples we can name RRM, Myb, bHLH, Sox, homeobox, forkhead, APSES, HSF, AT hook, copper fist, and CAAT-binding, amongst others. Although these proteins were identified in low numbers of copies, they play important functional roles, such as fungal adaptation to host and environment described in the group of forkhead proteins (Wang et al., 2015) and fungal differentiation and secondary metabolism (Son et al., 2020). In addition, diverse families have been proposed as specific to fungi, such as APSES and copper fist, because they were found exclusively in fungal genomes (Shelest, 2017).

In summary, the TF families identified in both *Penicillium* species are very similar to the fungal genomes used as reference; where three families (Zn2Cys6, C2H2 ZF, and RRM) include around 75% of the total of the DNA-binding domains identified in *P. echinulatum* and *P. oxalicum* genomes. When the role of these core families was explored in the fungi used as reference, central processes were found, such as carbon and nitrogen metabolism, amino acid and vitamin synthesis, growth and development (Scazzocchio, 2000), and fungal differentiation and secondary metabolism, among others.

### 3.3. General Properties of the Regulatory Network

The GRN in the *Penicillium* species was inferred considering orthology information and curated regulatory interactions from the model organisms *A. nidulans, N. crassa*, and *S. cerevisiae*. A regulatory interaction was defined as a TF-TG relation, where TF is the regulator gene and TG is the target gene. For each regulatory interaction of the model fungi, when orthologous were found for both TF and TG in the *Penicillium* species, a new TF-TG relation for the respective *Penicillium* species was created. Besides the TF-TG orthology, we also performed TFBSs predictions for each TF-TG identified. Based on these data, we constructed the global regulatory networks of *P. echinulatum* and *P. oxalicum*.

**Figure 4:**
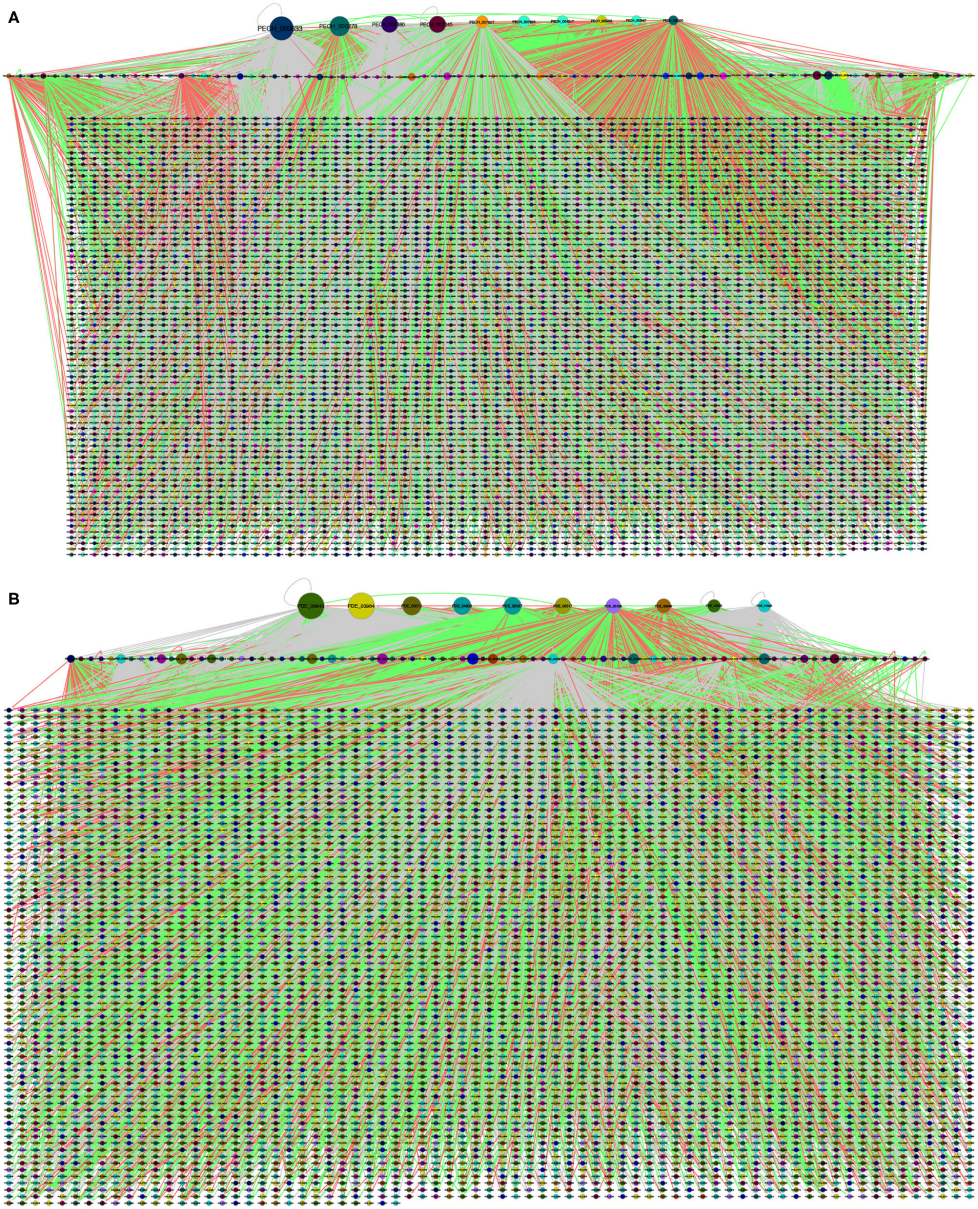
Global regulatory networks of **(A)** *P. echinulatum* 2HH and **(B)** *P. oxalicum* 114-2. Each community is represented in a different color. The node size is proportional to the output degree. In the first level, we show the hub nodes, in the second level the remained TFs and finally, the target nodes.

Therefore, the GRN of *P. echinulatum* shown in Figure 4A contains 5,862 nodes and 21,184 regulatory interactions. Based on the TF-TG orthology, 96 TFs and 5,853 TGs were identified in the GRN, and that covers 71.7% of the *P. echinulatum* proteome. From these 96 TFs, 87 are also TGs; i.e., they could be self-regulated or regulated by another TF. The vast majority of regulatory interactions inferred for *P. echinulatum* came from orthology related to only one model fungus. This group totalized 21,067 interactions, of which 5,962 resulted from *A. nidulans*, 10,723 derived from *N. crassa*, and 4,382 originated from *S. cerevisiae*. Another group of 115 regulatory interactions inferred for *P. echinulatum* came from orthology found in two model fungi, of which 90 interactions came from curated regulatory interactions found in both *A. nidulans* and *N. crassa*; other three interactions were derived from *A. nidulans* and *S. cerevisiae*; and 22 interactions came from *N. crassa* and *S. cerevisiae*. Finally, two interactions were inferred by orthology of curated regulatory interactions that are conserved in the three model fungi.

A similar behavior was observed in the inference of the *P. oxalicum* GRN shown in Figure 4B that contains 5,528 nodes and 16,775 regulatory interactions, of which 99 are TFs and 5,516 are TGs. From these 99 TFs, 86 are also TGs. This inferred GRN covers 55.4% of the *P. oxalicum* proteome. The vast majority of regulatory interactions also came from orthology related to only one model fungus. This group totalize 16,685 interactions, of which 6,099 resulted from *A. nidulans*, 8,677 derived from *N. crassa*, and 1,909 originated from *S. cerevisiae*. The second group of 90 regulatory interactions came from orthology found in two model fungi, of which 87 interactions came from *A. nidulans* and *N. crassa*; two interactions derived from *A. nidulans* and *S. cerevisiae*; and one interaction came from *N. crassa* and *S. cerevisiae*. Finally, no interactions were inferred for *P. oxalicum* by orthology of curated regulatory interactions that are conserved in the three model fungi.

### 3.4. Topological Properties of the Regulatory Network

In order to characterize the structure of GRN of *Penicillium* species, the general structure of both networks was analyzed in Table 1. We identified that the networks are structured into a single giant component in which there is a path between each pair of nodes.

**Table 1:** General properties of the regulatory networks.

	***P. echinulatum* 2HH**	***P. oxalicum* 114-2**
Total of nodes	5,862	5,528
Interactions	21,184	16,775
Auto-regulations	23	19
Positive edges	8,078	6,901
Negative edges	4,697	3,952
Unknown edges	8,409	5,922
Average degree	7.2	6.0
Connected components	1	1
Giant component	5,862	5,528
Maximum out degree	2,502 (PECH_000633)	1619 (PDE_06843)
Maximum in degree	24 (PECH_007435)	24 (PDE_00087)
Communities	20	19

Taking these data into account, we identified that 1,404 nodes in *P. echinulatum* and 1,776 nodes in *P. oxalicum* are regulated by only one TF, i.e., they have an input degree of 1; while the BrlA (PDE_00087) and its orthologous PECH_007435 are regulated by 24 TFs in *P. oxalicum* and *P. echinulatum*, respectively, making them the most regulated genes. BrlA (PDE_00087), identified as a member of the C2H2 transcription factor family, plays a key role in regulating conidiation, affecting also the regulation of secondary metabolism and the expression of cellulase genes (Qin et al., 2013). Concerning the output degree, the most connected nodes are influencing 2,502 and 1,619 nodes, representing 42 and 29% of total nodes in the GRNs of *P. echinulatum* and *P. oxalicum*, respectively.

We also found that the highest clustering coefficient is 1, meaning that nodes whose neighbors are connected between them are forming complete graphs. We identified this property for 78 nodes in *P. echinulatum* and 36 in *P. oxalicum*, suggesting the existence of substructures, such as triangles or more complex motifs. On the other hand, 2,143 nodes in *P. echinulatum* and 2,902 in *P. oxalicum* have a clustering coefficient equal to 0; whereas the average clustering coefficient for the network was 0.20 for *P. echinulatum* and 0.13 for *P. oxalicum*. This result indicates that neighbors have, on average, $\frac{1}{5}$ of connections for *P. echinulatum* and $<\frac{1}{5}$ for *P. oxalicum*. In this regard, when the clustering coefficient is large, a small world network structure can be described, which is not the case analyzed here.

### 3.5. Identification of Communities on the GRN

In order to identify the most connected elements, we analyzed the network in terms of communities. A community was defined as a subset of nodes densely connected in comparison with the rest of the network, and its identification may help to discover relations not previously identified (Radicchi et al., 2004). Based on this approach, we identified 20 communities in the GRN of *P. echinulatum*, where the largest one contains 1,170 nodes and the smallest one contains 38 nodes; while for the GRN of *P. oxalicum*, 19 communities were identified, where the largest one contains 706 nodes and the smallest one contains 23 nodes.

**Figure 5:**
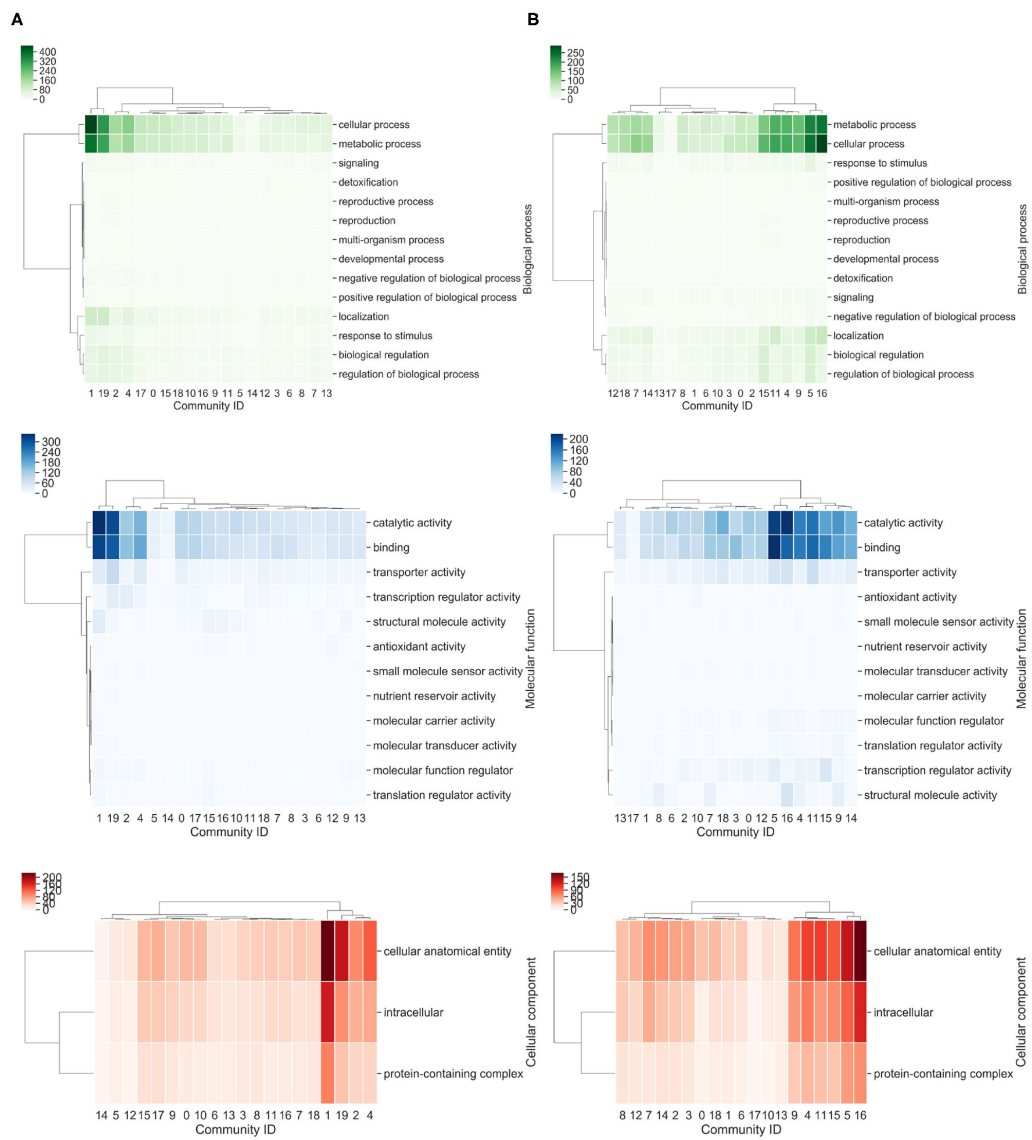
Heatmap of Gene Ontology terms associated with communities. **(A)** *P. echinulatum* 2HH and **(B)** *P. oxalicum* 114-2. The richest GO terms for each community were identified. A hierarchical clustered based on Euclidean distance measure and Ward's method for linkage analysis was achieved. Each row represents the GO term and each column represents community ID.

To determine the most abundant function, each community was analyzed with the Gene Ontology (GO) terms enrichment (Figure 5). Based on this approach, we identified that community 4 for *P. oxalicum* and 19 for *P. echinulatum* are enriched of genes related to cellular process, metabolic process and localization. On the other hand, communities 5, 11, and 16 in *P. oxalicum* and the community 19 in *P. echinulatum* are the most diverse, where the most abundant functions are catalytic activity, binding, and transporter activity (Figure 5).

Therefore, we found that communities 19 of *P. echinulatum* and 16 of *P. oxalicum* share a high proportion of orthologous proteins, and similarities at molecular function level. In contrast, community 4 of *P. oxalicum* contains orthologs from various communities of *P. echinulatum*, such as 19, 17, and 4. In addition, communities 3 and 11 in *P. echinulatum* and community 2 in *P. oxalicum* contain genes involved in the xylanolytic and cellulolytic transcriptional activator systems, among others. In summary, we not only identified that both *Penicillium* species share similar communities at sequence and functional levels, but also, particular communities in each species, which shows their diversity.

### 3.6. Mining the Regulatory Network

In order to identify the most connected nodes associated with the network, hubs were identified. Hence, a hub was defined as a TF with connections to many other nodes, i.e., a large output degree. Therefore, we showed the ten top hubs in *P. echinulatum* and *P. oxalicum* (Table 2).

**Table 2:** Top 10 of hub nodes.

***P. echinulatum*** **2HH**		***P. oxalicum*** **114-2**			
**Transcription factor**	**Out degree**	**Transcription factor**	**Out degree**	**TF family**	**Regulated process**
PECH_000633 (CpcA^*^)	2502	PDE_08488 (CpcA^*^)	^#^	bZIP	Protein synthesis.
PECH_000278 (COL-26^**^)	2011	PDE_04455 (COL-26^**^)	^#^	Zn2Cys6	Starch utilization.
PECH_007045 (FF-7^**^)	1611	PDE_06843 (FF-7^**^)	1619	Zn2Cys6	Sexual development.
PECH_001380 (AmyR^*^)	1595	PDE_03964 (AmyR^*^)	1612	Zn2Cys6	Starch utilization.
PECH_007907 (LreB^*^)	1053	PDE_08612 (LreB^*^)	1046	GATA	Blue-light responsive differentiation.
PECH_007823 (AtfA^*^)	957	PDE_04903 (AtfA^*^)	972	bZIP	Oxidative and osmotic stress-responsive genes.
PECH_004317 (FlbB^*^)	932	PDE_06387 (FlbB^*^)	950	bZIP_YAP	Conidiophore development.
PECH_005206 (RES-1^**^)	844	PDE_06517 (RES-1^**^)	857	C2H2	Endoplasmic reticulum stress response.
PECH_006987 (MetZ^*^)	723	PDE_05199 (MetZ^*^)	745	bZIP	Sulfur metabolism.
PECH_000930 (CCG-8^**^)	715	PDE_09849 (CCG-8^**^)	723	TF_Opi1	Biological processes (Clock-controlled gene).
		PDE_02029 (NsdD^*^)	609	GATA	Conidiation and cell wall stress resistance.
		PDE_01826 (GAL4^***^)	574	Zn2Cys6	Galactose-inducible genes.

TFs are ordered according to the out degree value. Proteins from columns 1 and 3 are orthologous. Gene names are shown in brackets according to the orthologous proteins of

* *A. nidulans*.

** *N. crassa*.

*** *S. cerevisiae*.

# *Incorrect annotation, orthology not identified by our approach. See alignment in Supplementary Data Sheet 1*.

In the GRN of *P. echinulatum*, the most connected node is PECH_000633 that consists of 2,502 inferred interactions. This bZIP transcription factor is orthologous to CpcA of *A. nidulans*, CPC-1 of *N. crassa*, and Gcn4p of *S. cerevisiae*. In *S. cerevisiae*, Gcn4p stimulates the transcription of 12 different pathways related genes, and also genes encoding various aminoacyl-tRNA synthetases and pathway-specific activators. This cross-pathway regulatory network of amino acid biosynthesis is known as GAAC in yeast and CPC in *Neurospora* and *Aspergillus* (Hoffmann et al., 2001; Hinnebusch, 2005). Recently, Gcn4p was described as a central regulator of protein synthesis, holding a key role in stress response and longevity. The reduction of the protein synthesis capacity by this regulator extends yeast lifespan (Mittal et al., 2017). The control of amino acid starvation by CpcA in *A. nidulans* also regulates sexual development, revealing a connection between metabolism and sexual development in filamentous fungi (Hoffmann et al., 2000). Therefore, PECH_000633 could be considered as a central regulatory protein associated with fundamental physiological processes, similar to Gcn4p, and CPC transcription factors. The conservation of this important regulatory system suggests subsequent studies regarding the fungal lifespan, given that longer lifetime is highly important to improve the production of cellulolytic enzymes by *Penicillium* spp.

The second most connected node of the *P. echinulatum* GRN is PECH_000278 with 2,011 regulatory interactions. This Zn2Cys6 binuclear cluster transcription factor is orthologous to COL-26 of *N. crassa*. In *N. crassa*, COL-26 is necessary for the expression of amylolytic genes and is required for the utilization of maltose and starch. This TF also acts as a regulator of glucose metabolism and its loss causes resistance to carbon catabolite repression, affecting integration of carbon and nitrogen metabolisms (Xiong et al., 2017). Several regulatory interactions of biotechnological interest were inferred for PECH_000278, covering the major cellulolytic enzymes of *P. echinulatum*: intracellular β-glucosidase (PECH_005648), cellobiohydrolase (PECH_007386), endoglucanase EGL1 (PECH_009029), β-glucosidases (PECH_005824 and PECH_002471), and xylanases (PECH_006995 and PECH_007282). In addition, regulatory interactions related to the major amylolytic genes were inferred for PECH_000278, including α-amylases (PECH_008724 and PECH_000987), α-glucosidases (PECH_001379 [GH31] and PECH_005310 [GH15]), and a lytic starch monooxygenase (PECH_007113). These inferred regulatory interactions suggest that PECH_000278 is a promising target for industrial strains improvement, due to its role in sugar metabolism regulation.

In *P. oxalicum*, our approach could not identify the orthologous of these two most connected TFs, highlighted in the GRN of *P. echinulatum*. The blastp querying PDE_08488 against UniprotKB showed the following results: AN3675-CpcA of *A. nidulans* (*e*-value: 3.4e-36; ident.: 46.0%; coverage length: 100%, NCU04050-CPC-1 of *N. crassa* (*e*-value: 1.3e-16; ident.: 31.7%; coverage length: 100%) and YEL009C-GCN4 of *S. cerevisiae* (*e*-value: 2e-10; ident.: 29.9%; coverage length: 100%). The results showed that the length of PDE_08488 is quite larger when compared to the reviewed CpcA, CPC-1, and GCN4. Our results are in agreement with the previously identified incorrect annotation of CpcA in *P. oxalicum* (Pan et al., 2020). In a blastp using PDE_04455 as query against UniprotKB, the best hit is PENSUB_363 (*P. subrubescens*) (*e*-value: 0.0; ident.: 58.8%; coverage length: 88.1%). In PDE_04455, only the domain IPR007219 was identified, different from their homologs in closely related *Penicillium* species. The blastp result also suggests that its transcription start site is probably misannotated and possibly there is a Zn2Cys6 binuclear cluster upstream of PDE_04455, as occurs in *P. subrubescens* and *P. echinulatum* (PECH_000278). These results suggest that both PDE_08488 and PDE_04455 are misannotated in *P. oxalicum*.

As shown in Table 2, the third most connected node in the GRN of *P. echinulatum* is orthologous to the first most connected node in the GRN of *P. oxalicum*. The subsequent most connected nodes in the two GRNs follow this orthological relationship, up to the tenth most connected node in the GRN of *P. echinulatum* that corresponds to the eighth most connected node as its ortholog in the GRN of *P. oxalicum*.

The most connected node (PDE_06843) in the GRN of *P. oxalicum*, is a Zn2Cys6 binuclear cluster transcription factor, ortholog to the third most connected node (PECH_007045) in the GRN of *P. echinulatum* and also ortholog to FF-7 in *N. crassa*. In *N. crassa*, the female fertility-7 (FF-7) is required for initiation of sexual development, and Δ*ff-7* mutant does not produce protoperithecia and perithecia as well as ascospores (Carrillo et al., 2017). In *P. oxalicum* HP7-1, it was demonstrated that POX09752, ortholog of PDE_06843 in *P. oxalicum* 114-2, is involved in the regulation of raw-starch-digesting enzymes (Zhang et al., 2019). This is consistent with the inferred regulatory interactions where PDE_06843 regulates one α-amylase encoding gene PDE_04683 (GH13-5) in *P. oxalicum*; and PECH_007045 regulates two α-amylase encoding genes PECH_000987 (GH13-5) and PECH_000986 (GH13-1) in *P. echinulatum*.

The next hub node refers to AmyR orthologs, also belonging to the Zn2Cys6 binuclear cluster family. Its orthologs are PECH_001380 and PDE_03964 in *P. echinulatum* and *P. oxalicum*, respectively. AmyR has been described as a positive regulator of amylase encoding genes, involved in starch utilization in *Aspergillus* species (Benocci et al., 2017). Its orthologs were found in several *Ascomycetes*, and its function has been substantially studied in *P. oxalicum*, in which the Δ*amyR* mutant resulted in a substantial increase of cellulase activity (Li et al., 2015).

In this regard, the Δ*amyR* mutant of *P. oxalicum* was reported as deficient for transcribing the major raw starch-digesting glucoamylase gene *gluA* (PDE_09417) when grown on cellulose. The lack of AmyR also affects a wide range of CAZymes involved in the starch metabolism (Li et al., 2015). In the GRN of *P. oxalicum*, AmyR exhibited 1,612 inferred regulatory interactions, including some of the major downregulated CAZymes involved in the starch metabolism of the Δ*amyR* mutant: PDE_01201 α-amylase Amy13A (GH13-1), PDE_09417 glucoamylase GluA (GH15), PDE_03966 α-glucosidase (GH31), and PDE_01354 lytic starch monooxygenase (AA13). In addition, our inferred regulatory interactions also include some of the major cellulase encoding genes, upregulated in the Δ*amyR* mutant: PDE_07124 cellobiohydrolase (GH6), PDE_09226 endoglucanase (GH5-5), and PDE_04251 β-glucosidase (GH3).

In *P. echinulatum* GRN, 1,595 regulatory interactions were predicted for AmyR, including all ortholog genes related to starch and cellulose metabolisms described for *P. oxalicum*. As discussed above, our results are in agreement with the reported regulatory role of AmyR in *Aspergillus* spp. and *P. oxalicum*, affecting positively the expression of starch-related enzymes and negatively the expression of cellulose-related enzymes.

Although various TFs were identified as hubs in the GRNs, additional transcription factors not identified in the top ten of highly connected nodes according to the metric of output degree are relevant to the regulation of the cellulolytic system, such as XlnR, ClrB, and CreA. ClrB is a Zn2Cys6 zinc finger transcription factor, known for its role in the positive regulation of cellulolytic enzymes in filamentous fungi. In *P. oxalicum* 114-2, we identified that ClrB (PDE_05999) could regulate 472 genes; and experimental evidence showed its positive regulating role of major cellulolytic enzymes (Li et al., 2015). Inferred regulatory interactions found in our GRN of *P. oxalicum* are in agree with previous experimental evidence, including cellobiohydrolases (PDE_07124, PDE_07945, and PDE_05445), endoglucanases (PDE_09226, PDE_03711, PDE_05193, PDE_07929, and PDE_02886), LPMOs (PDE_06768 and PDE_01261), and BGL2 (PDE_00579). Similarly, in *P. echinulatum*, ClrB (PECH_005720) could regulate 479 genes, including cellobiohydrolases (PECH_006365, PECH_008028, and PECH_007386), endoglucanases (PECH_009029, PECH_007371, and PECH_005815), LPMOs (PECH_008064 and PECH_007161), and BGL2 (PECH_005648).

In contrast, CreA plays a key role in the carbon catabolite repression regulatory system which prevents wasting energy on the production of extracellular enzymes, as well as metabolic routes that are not needed. Therefore, this C2H2 transcription factor represses the expression of cellulolytic genes. In *P. oxalicum* 114-2, CreA (PDE_03168) represses the expression of major cellulases (Li et al., 2015). These previous experimental evidences are in accordance with the regulatory interactions found in our GRN of *P. oxalicum* 114-2 (464 regulated genes), including cellobiohydrolases (PDE_07124 and PDE_07945), endoglucanases (PDE_09226, PDE_03711, PDE_05193, and PDE_07929), LPMOs (PDE_06768 and PDE_01261), and BGL2 (PDE_00579). Similarly, in the GRN of *P. echinulatum* 2HH we found that CreA (PECH_004563) regulates 457 genes; where interactions covering cellobiohydrolases (PECH_006365 and PECH_007386), endoglucanases (PECH_009029 and PECH_007371), LPMOs (PECH_008064 and PECH_007161), and BGL2 (PECH_005648), were identified.

**Figure 6:**
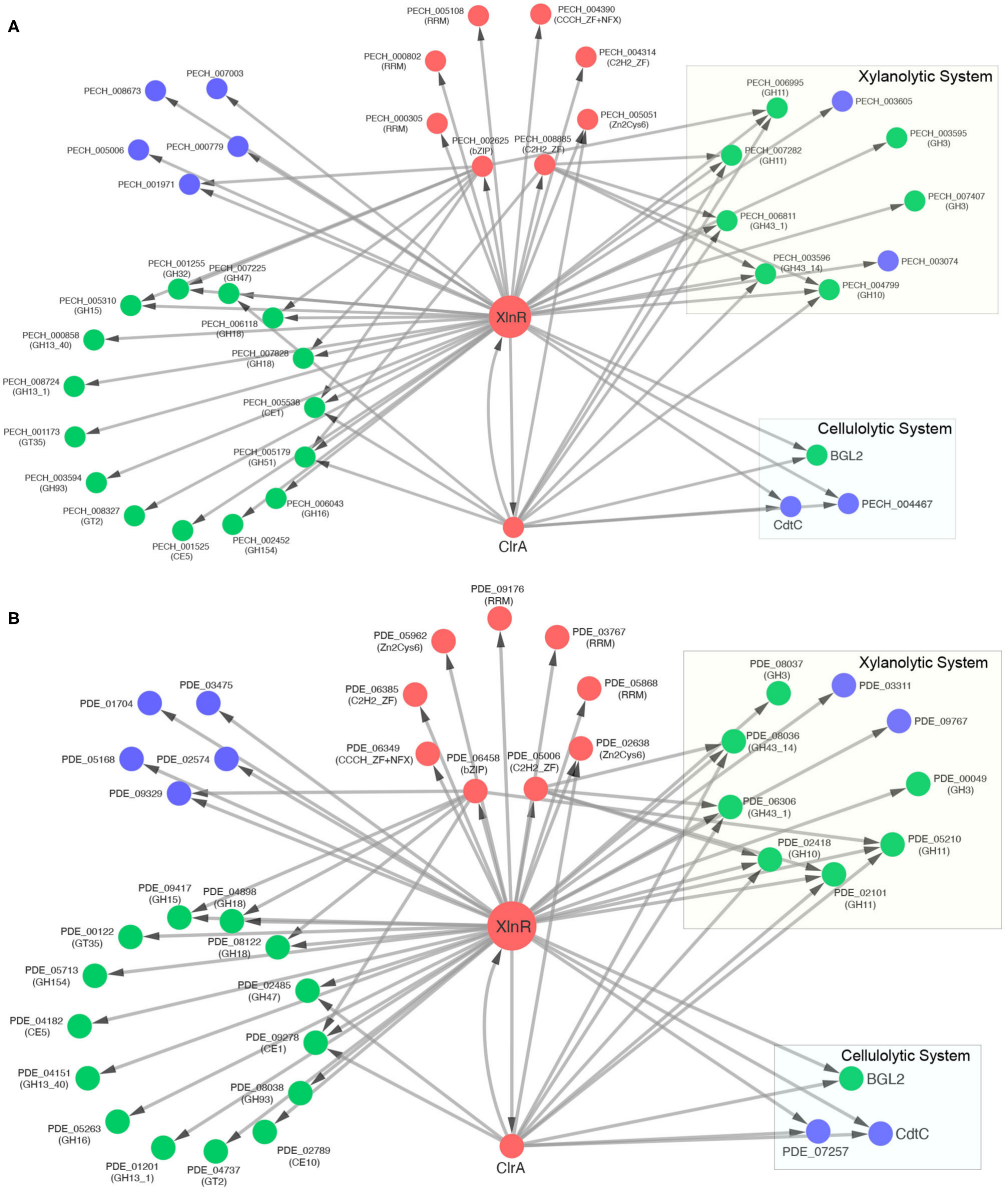
Regulatory networks of XlnR-TGs containing only CAZymes, TFs, and sugar transporters. **(A)** *P. echinulatum* 2HH and **(B)** *P. oxalicum* 114-2. CAZymes are colored in green, TFs in red, and sugar transporters in blue. Rectangles highlight the major CAZymes and sugar transporters involved in xylanolytic and cellulolytic systems. Arrows point the regulatory direction. The regulatory relationship between XlnR and ClrA is bidirectional.

The xylanolytic and cellulolytic transcriptional activator XlnR of *P. echinulatum* contains a repetitive sequence in the coding region, resulting in fragments placed in two different scaffolds of the WGS. However, the complete protein was used in this study, once this gene was sequenced and deposited at GenBank (accession number: MT676450 and locus tag: PECH_002137). This TF comprehends 250 inferred regulatory interactions in *P. echinulatum* while its ortholog PDE_07674 in *P. oxalicum* include 251 interactions. As expected, quite a few regulatory interactions involving xylanolytic enzymes were inferred for both species, including the major xylosidases of the CAZy families GH3 (2 genes) and GH43 (2 genes), the main xylanases of families GH10 (1 gene) and GH11 (2 genes) and the major xylose transporters (2 genes). The major XlnR-dependent proteins involved in the xylanolytic and cellulolytic systems are detailed in Supplementary Table 3 and presented in Figure 6.

In order to provide accuracy to the TF-TG relationships inferred for XlnR, predictions of TFBSs were performed in the upstream sequences of each TG. Our approach used the PWM matrix of XLR-1 (M02621_2.00), the XlnR ortholog found in *N. crassa*. For the major β-xylosidase (GH3), despite a significant difference in the composition of nucleotides, two conserved motifs were predicted in both species: XlnR-PECH_007407 predicted (CGGCTAATA) and (CGGTTACGT); XlnR-PDE_00049 predicted (TGTATATAT) and (TATATATAC). For both fungi, no motifs were predicted for the TF-TG relationships of the second β-xylosidase (GH3): XlnR-PECH_003595 and XlnR-PDE_08037. For the xylanase of the GH10 family, the same motif (CGGCTAAAA) was identified for both fungi relationships: XlnR-PECH_004799 and XlnR-PDE_02418. The DNA-binding site associated to XlnR in *Penicillium* was predicted by using the PWM matrix of XLR-1 (M02621 2.00), This weight matrix identified diverse probable sites recognized by this TF, such as the sequence CGGCTAATA and CGGTTACGT of XlnR-PECH_007407 and TGTATATAT and TATATATA for XlnR-PDE_00049. One of these motifs is similar to the identified in the *gsn* gene 5'-flanking region of *N. crassa* (GGCTGA) (Gonçalves et al., 2011). However, we must remember that our approach considers a PWM matrix that is a model for the binding specificity of a TF and can be used to scan a sequence for the presence of DNA sites that are significantly more similar to the PWM than to the background (Stormo, 2013). The complete prediction dataset is provided in Supplementary Table 3.

For the first xylanase of the GH11 family, the motif (CGGGTAAAT) was predicted for XlnR-PECH_006995 in *P. echinulatum*, while for XlnR-PDE_05210 in *P. oxalicum* no motifs were predicted. Aligning these promoter regions, we observed that the last “T” of the predicted motif in *P. echinulatum* is an “A” in *P. oxalicum*. In contrast, for the second xylanase of the GH11 family, there was found a motif (CGGATAAAT) only for XlnR-PDE_02101 in *P. oxalicum*, while for XlnR-PECH_007282 it was not predicted. For the β-xylosidase (GH43-14) a XlnR binding-site (CGGTTAACG) was predicted for PECH_003596 in *P. echinulatum*, while in *P. oxalicum* this binding-site was not found for PDE_08036. In contrast, for the β-xylosidase (GH43-1) XlnR binding-sites were predicted for both species. In *P. echinulatum*, (CGGTTAATT) was predicted for PECH_006811 and, in *P. oxalicum*, the motif (CGGCTAAAC) was predicted for PDE_06306. For the xylose transporters XlnR binding motifs were not found in both species.

Regulatory interactions for the major cellulolytic enzymes were not inferred for XlnR. Nevertheless, XlnR includes inferred regulatory interactions for some regulators of cellulase expression previously described. In *P. oxalicum*, ClrA (PDE_04046) is a Zn2Cys6 positive regulator of cellulase transcription (Liu et al., 2013c), orthologous to PECH_001863, CLR-1 and ClrA in *P. echinulatum, N. crassa*, and *Aspergillus* spp., respectively. Our approach found a XlnR binding-site (ACTTATACT) in the upstream region of *clrA* in *P. oxalicum*, while no motifs were predicted for *clrA* in *P. echinulatum*.

Cellobiose is the primary end product generated from cellulose degradation by cellulolytic enzymes. It has been shown that cellulase production is induced by cellobiose and other cellodextrins in many species of fungi (Aro et al., 2005). We inferred a regulatory interaction of XlnR to the major intracellular β-glucosidase BGL2 (PECH_005648/PDE_00579). However, no XlnR binding-sites were predicted for *bgl2* in both *P. oxalicum* and *P. echinulatum*. In *P. oxalicum*, BGL2 has been previously identified as a negative regulator of cellulase expression, considering that the Δ*bgl2* mutant raised remarkably the secretion of cellulolytic enzymes (Chen et al., 2013).

Besides BGL2, cellulase expression is also affected by cellodextrin transporters. Two major XlnR-dependent cellodextrin transporters were inferred for both fungi. The first cellodextrin transporter CdtC (PDE_00607) increased cellulase production in *P. oxalicum* mutant when overexpressed, denoting that CdtC played a positive regulatory role (Li et al., 2013). CdtC is ortholog to PECH_005610 in *P. echinulatum*, LacpB-CltB in *A. nidulans* (Dos Reis et al., 2016; Fekete et al., 2016) and CDT-1 in *N. crassa* (Znameroski et al., 2012). A XlnR binding-site (GGTATATAA) was predicted for *cdtC* in *P. echinulatum*, while in *P. oxalicum* this binding-site was not found. However, when both promoter regions (PECH_005610 and PDE_00607) were aligned, the same motif was found in *P. oxalicum*. We suggest that this RSAT misprediction could be a false negative. Further, the orthologs of *N. crassa* cellodextrin transporter CDT-2 (Znameroski et al., 2012) were also inferred as XlnR-dependent in the GRNs of *P. echinulatum* (PECH_004467) and *P. oxalicum* (PDE_07257). Lastly, for CDT-2 orthologs, no XlnR binding-sites were predicted.

Our results suggest that XlnR may play a background regulatory role in the expression of the cellulolytic system, acting through the cellodextrin induction system. On the other hand, XlnR plays a key role in positive regulation of the xylanolytic system. This remarkable finding of our study suggests that the XlnR-dependent cellulolytic and xylanolytic regulatory systems are probably conserved in both *P. oxalicum* and *P. echinulatum*. Our results are in consonance with *P. oxalicum* experimental evidence previously reported and discussed below.

The Δ*xlnR* mutant of *P. oxalicum* revealed low expressions for cellobiohydrolase *cbh1* (PDE_07945) and endoglucanase *eg2* (PDE_09226) transcripts; and no expression for xylanase *xyn1* (PDE_08094) when compared with the wild-type in cellulose growth medium. Besides other experimental evidence, it was suggested that XlnR is a general TF that facilitates the induction of cellulase expression under cellulose growth conditions. XlnR might participate in a transcriptional cascade that regulates the expression of genes coding for cellulolytic enzymes in *P. oxalicum* (Li et al., 2015).

The overexpression of *xlnR* was expected to upregulate the expression of the major extracellular β-xylosidase xyl3A (PDE_00049) in *P. oxalicum*. This was confirmed by OE*xlnR*Δ*laeA* mutant, which induced a remarkable 28.5 fold increase in the expression of *xyl3A* in relation to the wild-type. This result also showed that the regulation of *xyl3A* is not LaeA-dependent as the regulatory system of most of the cellulases or hemicellulases (Li et al., 2016).

In summary, the GRNs of *P. echinulatum* 2HH and *P. oxalicum* 114-2 showed similar components, such as those TFs identified as hubs according to the metric of output degree. In Table 2, the hub nodes were ordered according to TF orthology to highlight the high similarity between the two GRNs. Similar to the orthology identified among the hub nodes of both GRNs, most TGs regulated by each TF also preserve the orthology relationship in both GRNs. For example, 1526 TGs regulated by AmyR in *P. echinulatum* have their respective orthologous regulated by AmyR in *P. oxalicum*, while 69 are unique to the GRN of *P. echinulatum* and 86 are unique to the GRN of *P. oxalicum*. In this regard, both GRNs are highly similar, considering that both share mainly ortholog TFs and regulatory relationships for mainly ortholog TGs.

## 4. Conclusions

Our reconstructed network is a valuable resource of regulatory interactions occurring within *Penicillium* spp., and it may integrate with global expression data available for these fungal organisms in order to improve global interaction data models. In this regard, we found a group of proteins shared among the two *Penicillium* and three model fungi, involved in transcription and rRNA processing, i.e., genetic information flow. In addition, we demonstrate, through our analysis, the existence of large protein sets devoted to regulate gene expression in these fungal systems, where three families (Zn2Cys6, C2H2 ZF, and RRM) comprehend around 75% of the total of the DNA-binding domains identified in both fungal genomes. These genes are involved in regulation of central processes, carbon and nitrogen metabolism, amino acid and vitamin synthesis, growth and development, among others. Concerning the GRN, we found similar topological properties identified in other biological networks, highlighting the existence of at least ten global regulators. To name but a few of them in *P. echinulatum*, PECH_000633 could be considered fundamental in control of amino acid starvation, longevity and sexual development; PECH_000278 could be highly involved in carbon and nitrogen metabolisms; and PECH_007045 could be involved in the regulation of raw-starch-digesting enzymes. Finally, we explore diverse identified modules, such as the expression of cellulolytic and xylanolytic systems, where XlnR plays a key role in positive regulation of the xylanolytic system. It also regulates positively the cellulolytic system by acting indirectly through the cellodextrin induction system. This remarkable finding of our study suggests that the XlnR-dependent cellulolytic and xylanolytic regulatory systems are probably conserved in both *P. oxalicum* and *P. echinulatum*. Our results are in consonance with *P. oxalicum* experimental evidence previously reported. Finally, we explored the functional congruency on the genes clustered in terms of communities where the genes related to cellular nitrogen, compound metabolic process and macromolecule metabolic process were the most abundant.

## Data Availability Statement

All datasets presented in this study are included in the article/Supplementary Material or available in the FunRegulation project at Git http://www.github.com/alexandrelenz/funregulation.git.

## Author Contributions

All authors listed have made a substantial, direct and intellectual contribution to the work, and approved it for publication.

## Conflict of Interest

The authors declare that the research was conducted in the absence of any commercial or financial relationships that could be construed as a potential conflict of interest.

## Supplementary Material

Supplementary Table 1 Distribution of transcription factor domains.

Supplementary Table 2 Curated transcriptional regulatory interactions in model fungi.

Supplementary Table 3 Major XlnR-dependent proteins involved in the xylanolytic and cellulolytic systems

Supplementary Data Sheet 1 Alignment of incorrect annotated proteins of Penicillium oxalicum 114-2.
