# Supplementary material for: Gene Regulatory Networks of Penicillium echinulatum 2HH and Penicillium oxalicum 114-2 Inferred by a Computational Biology Approach
Source: Front Microbiol. 2020 Oct 27;11:588263. doi: 10.3389/fmicb.2020.588263 (PMC7652724; doi:10.3389/fmicb.2020.588263)
Supplement: Supplementary Data Sheet 1 — Alignment of incorrect annotated proteins of Penicillium oxalicum 114-2. [file Data_Sheet_1.PDF]

CLUSTAL O(1.2.4) multiple sequence alignment

S7ZXM4 - **PDE_04455** - *Penicillium oxalicum (strain 114-2)*
Q5B705 - **CPCA** – *Aspergillus nidulans (strain FGSC A4)*
P11115 - **cpc-1** - *Neurospora crassa (strain OR74A)*
P03069 - **GCN4** - *Saccharomyces cerevisiae (strain S288c)*

```
TR|S7ZXM4|S7ZXM4_PENO1 MESPFTGPLNFSIASRSSSSPQIIISPIDFTSFTTDSSQQPWPPTPPSTQPLASNSINSN 60
TR|Q5B705|Q5B705_EMENI ------------------------------------------------------------
SP|P11115|CPC1_NEUCR   ------------------------------------------------------------
SP|P03069|GCN4_YEAST   ------------------------------------------------------------


TR|S7ZXM4|S7ZXM4_PENO1 NSSSNSSLQDFVLYPTPPASQPQPPPRDSRLLAPPSTTLKPSALQPFLAQHQPRRHTLSL 120
TR|Q5B705|Q5B705_EMENI ------------------------------------------------------------
SP|P11115|CPC1_NEUCR   ------------------------------------------------------------
SP|P03069|GCN4_YEAST   ------------------------------------------------------------


TR|S7ZXM4|S7ZXM4_PENO1 QLQRYLQQQLSSGLPISDPRVAKLARSFAHWSHSNSSSYAQQPHAAAVPSPLRPQSGAQF 180
TR|Q5B705|Q5B705_EMENI ------------------------------------------------------------
SP|P11115|CPC1_NEUCR   ------------------------------------------------------------
SP|P03069|GCN4_YEAST   ------------------------------------------------------------


TR|S7ZXM4|S7ZXM4_PENO1 IPTLKKPYTTAYRRTMSAPGFEGTHLHHPSVPVPDSSPLAHTHATELDAALFALPSAGDT 240
TR|Q5B705|Q5B705_EMENI ---------------MSTPNIPH----QGEFGCPGSFWL--GLRTNLDDPEFF----DFT 35
SP|P11115|CPC1_NEUCR   ------------------------------------------MFSELDLLDFATFDGGAT 18
SP|P03069|GCN4_YEAST   -------------------------------------------MSEYQPSLFALNPMGFS 17
                                                                   :: :   *     . :

TR|S7ZXM4|S7ZXM4_PENO1 AGLGSPFDIGQI------------HLDTDV---NAFSPGAPTDTVSPKDVMFD--A--S- 280
TR|Q5B705|Q5B705_EMENI EGFGEDFTDPTMLSPH--------LVPTGIMASKDSLGDVPAGTVSPSDLFMD-----A- 81
SP|P11115|CPC1_NEUCR   -------TEAAFASPA--------NQTYDL-SSSVPSSVSNMGTVSPQELLLH--EPYL- 59
SP|P03069|GCN4_YEAST   PLDGSKSTNENVSASTSTAKPMVGQLIFDKFIKTEEDPIIKQD--TPSNLDFDFALPQTA 75
                                  .                .    .        .  :*.:: :.
```

```
TR|S7ZXM4|S7ZXM4_PENO1 SVP------PSATFTDLSTPLL--ESPGAFSSGPSPMFTDLDL--LGPNDWTPLFNDAAA 330
TR|Q5B705|Q5B705_EMENI SAP------PSTSFTDLSTPSF--DSPGYFSQDTSPVFGADLDLAPGHEEWAPLFPSNDG 133
SP|P11115|CPC1_NEUCR   SAP------SSTALTALTSPSLFDGSPDFDTFDISPNFGHSDL--ENPDTWFSLFPDATP 111
SP|P03069|GCN4_YEAST   TAPDAKTVLPIPELDDAVVESFFS-----SSTDSTPMFEYEN-LEDNSKEWTSLFDNDIP 129
                       :.*          :       :        : . :* *        . . *  ** .

TR|S7ZXM4|S7ZXM4_PENO1 MDAFQS---LAMVAPLEETSKTEKPLPAQTPKLAASVQ----------SV---------- 367
TR|Q5B705|Q5B705_EMENI MSMPFDPTGLEIAAPVPAVK-AEPTV------SSPTVK----------PV---------- 166
SP|P11115|CPC1_NEUCR   LPQAQAQVQTQPQTQ-TQTEQQTQPLPELVQSVQPTVQP--TVEQTVHSVEASPATPSED 168
SP|P03069|GCN4_YEAST   VTTDDVSLADKAIESTEEVS----LVPSNLEVSTTSFLPTPVLEDAK---------LTQT 176
                       :                 ..     :         :.

TR|S7ZXM4|S7ZXM4_PENO1 -------SSPI-------SATGSSKPSSVAGIN-RP-RKELSPIA-FDPSDPVAAKRARN 410
TR|Q5B705|Q5B705_EMENI -------SSPARSP--TATSRSTTKHSTVAGVSARR-SKPLPPIK-YDESDPVAAKRARN 215
SP|P11115|CPC1_NEUCR   LEVLSPGSGHQRRK--SSVSPPSGRHSSVAGVGSRRRDKPLPPIIVEDPSDVVAMKRARN 226
SP|P03069|GCN4_YEAST   RKVKKPNSVVKKSHHVGKDDESRLDHLGVVAYNRKQRSIPLSPIV-PESSDPAALKRARN 235
                              *                    *.. . :     * **   : ** .* *****

TR|S7ZXM4|S7ZXM4_PENO1 TEAARKSRAKKLERQMSAEARISELQKQLADRDAIIANLQAQLEAQRQFA- 460
TR|Q5B705|Q5B705_EMENI TEAARKSRARKLERQGDMERRIAELSKELEETRQMVEFWKSQAQARARGA- 265
SP|P11115|CPC1_NEUCR   TLAARKSRERKAQRLEELEAKIEELIAERDR-------WKNLALAHGASTE 270
SP|P03069|GCN4_YEAST   TEAARRSRARKLQRMKQLEDKVEELLSKNYHLENEVARLKKLVGER----- 281
                       * ***:** :* :*  . * :: **  :           :     :
```

CLUSTAL O(1.2.4) multiple sequence alignment

S7ZFQ0 - **PDE_04455** - *Penicillium oxalicum (strain 114-2)*
A0A1Q5UN98 - **PENSUB_363** - *Penicillium subrubescens*
A0A0F7TSE6 - **PMG11_08231** - *Penicillium brasilianum*
Zn2Cys6 Domain highlighted in grey

```
TR|S7ZFQ0|S7ZFQ0_PENO1         ---------------------------------------------------------MRR 3
TR|A0A1Q5UN98|A0A1Q5UN98_9EURO MPQPTRNIIKWACDACKLRKVKCSSESPCSGCVAAGISCTFNKRPLVRGPRGLRAKTIRR 60
TR|A0A0F7TSE6|A0A0F7TSE6_PENBI MPQSTRKIGKWACDACKLRKVKCSSESPCSGCVTAGILCTFNKRPLVRGPKGLRAQTIRR 60
                                                                                        :**

TR|S7ZFQ0|S7ZFQ0_PENO1         ITATQQQHQQQQNRDPCE-DTVDLCERQ-------LSHANSGRRSNSPSPCNRGIPSSTT 55
TR|A0A1Q5UN98|A0A1Q5UN98_9EURO ITATQQQQQQQQ--QQQQEQDYGWCDIAGSPSGRRPGQITPARSSSTPSPSSDTHE-VTT 117
TR|A0A0F7TSE6|A0A0F7TSE6_PENBI ITATQQQQQQQQQQEQQEQQFYGWGSNSGSPGERRTGQTPPAMPSSTPSPSNDTYQ-VTT 119
                               *******:****  :  : :  .  .          .:   .  *.:***..      **

TR|S7ZFQ0|S7ZFQ0_PENO1         SDPPASTPERHAGDEPCRVSTSSLVLQLCVYRLRLFPVWPIVDVEEIMGSLHRDAGNLDA 115
TR|A0A1Q5UN98|A0A1Q5UN98_9EURO PGSNALSSTNRTSDEPSRTSTSSLVLRLCVYRLRLFPVWPIVDVEEVMGNIHRDPNNMDA 177
TR|A0A0F7TSE6|A0A0F7TSE6_PENBI PDSTTLPSISHNIDEPHRTSTSSMVLRLCVYRLRLFPVWPIVDVEEVMGNIHRDPDNMAA 179
                                .  :     :  *** *.****:**:*******************:**.:*** .*: *

TR|S7ZFQ0|S7ZFQ0_PENO1         YALATAIGAATIAQLKLDSPESGNMAMAASMEAECQGAIDHIRRKQQSSDLNINSIRVSF 175
TR|A0A1Q5UN98|A0A1Q5UN98_9EURO YALANAIGAATIAQLKLDSPENGDMATAASMEAECQRAIELLRKDGGDSTMNVNALRVSF 237
TR|A0A0F7TSE6|A0A0F7TSE6_PENBI YALANAIGAATIAQLKLDSPENGDMATAASMEAECQRAIELLRKDGGDSIMDINALRVSF 239
                               ****.****************.*:** ********* **: :*:.  .* :::*::****

TR|S7ZFQ0|S7ZFQ0_PENO1         FLHVYHENSAPGGPRSLLYLREAISIAQIMGLHRESSYVHLTTREQRMRQRIMSLLFVTE 235
TR|A0A1Q5UN98|A0A1Q5UN98_9EURO FLHVYHENRSPGGSRSLLYLREAITIAQIMGLHREATYSSLSTREQQMRQRILSLLFVTE 297
TR|A0A0F7TSE6|A0A0F7TSE6_PENBI FLHVYHENRSPGGSRSLLYLREAITIAQIMGLHREATYSSLSAREQQMRQRILSLLFITE 299
                               ******** :*** **********:**********::*  *::***:*****:****:**

TR|S7ZFQ0|S7ZFQ0_PENO1         RGVAMLHKLPVILQFNSRFVNLDESDDETHVLPAFKKLVTLFSLIDQSGVFASLHNSDEV 295
TR|A0A1Q5UN98|A0A1Q5UN98_9EURO RGVAMLHKLPVMLRYNARFINLDEADDESHVLPAFKKLVSLFLMFDQSGAFDILHTSDEI 357
TR|A0A0F7TSE6|A0A0F7TSE6_PENBI RGVAMLHKLPVILRYNARFINLDEADDESHVLPAFKKLMSLFLMFDQSGAFDMLHNSDEI 359
                               ***********:*::*:**:****:***:*********::** ::****.*  **.***:
```

```
TR|S7ZFQ0|S7ZFQ0_PENO1         --QPFDGISAINRGTFSSLQRQLREAAFQLRETNDIQMADIWVTTQWMQAILWRASMSRR 353
TR|A0A1Q5UN98|A0A1Q5UN98_9EURO QLPPYEGMHALNRNSFSLLQHQLREASIQLKETNDVQMADICVTTQWMQAILWRASMRRR 417
TR|A0A0F7TSE6|A0A0F7TSE6_PENBI QLPPYEGMPALNRNSFSLLQCQLREASIQPRETNVVQMADICVTTQWMQAILWRASMSRR 419
                                  *::*: *:**.:** ** *****::* :*** :***** *************** **

TR|S7ZFQ0|S7ZFQ0_PENO1         FATFDTPQQPVTSISHPIQIAKDFLEAMSRLPSSALEAHGPAM---------------EY 398
TR|A0A1Q5UN98|A0A1Q5UN98_9EURO -STLDSSQQHITSLSHPIQIAKDFLETMSHLPSSAIEAHGPAMVSNKNTLNEDESKLSEY 476
TR|A0A0F7TSE6|A0A0F7TSE6_PENBI -STLDSPQQQTTSLSHPIQIAKEFLETMSHLPSSAIEAHGPAM---------------EY 463
                                :*:*: **  **:********:***:**:*****:*******               **

TR|S7ZFQ0|S7ZFQ0_PENO1         KIFEIAKAVTDSVTTGLNDPTISIELSRDILHRLQSKLASFRGGNKTLLSLLHARISAAL 458
TR|A0A1Q5UN98|A0A1Q5UN98_9EURO KIYEIAKAVTDSVTTEFNGQPISLDIPREILLRLQSKLASCRGGNKVLLPLLHARISEAL 536
TR|A0A0F7TSE6|A0A0F7TSE6_PENBI KIYEIARAVVDSITTEFNGQPISLDIPRAILLRLQSKLASCRGGNKILLPLLHARISEAL 523
                               **:***:**.**:** :*.  **::: * ** ******** ***** ** ******* **

TR|S7ZFQ0|S7ZFQ0_PENO1         QDTNQRICNDSPPQDDRPVSSFSQGKHIYYPDHQIQPLPLVGPVPLRNFGTHGDMASRPG 518
TR|A0A1Q5UN98|A0A1Q5UN98_9EURO LRPSPRIYKIS---DYDPSSKPLSGERMLYSSQQSQSVPLSNPPSLRPAEIYEQQVNTEG 593
TR|A0A0F7TSE6|A0A0F7TSE6_PENBI LRPSPRDYKIS---DFGPSSKPLSRDTMPYSSQQPQILPLPSSPPLGYAEVYEEQGNLEG 580
                                  . *  : *   *  * *.  . . : * .:* * :** .   *     : :  .  *

TR|S7ZFQ0|S7ZFQ0_PENO1         QKSPHAMNDSLSTLHLMSWDHNLPEGFQSPHQDA-----LLSSTLADLDAAGAMELLFAN 573
TR|A0A1Q5UN98|A0A1Q5UN98_9EURO QELSQYTTDIVPTLRVVSWDHNIPDILLSSDQDPFNQAQVFQGSFGNIESAGTVEFLFAN 653
TR|A0A0F7TSE6|A0A0F7TSE6_PENBI RELPQDPTDNVSTLRVVTWDHNIPDILFSSDQYPFTQAQVFQGSFDNIDSTGTIEFLLNN 640
                               ::  :  .* : **::::****:*: : * .*       ::..:: :::::*::*:*: *

TR|S7ZFQ0|S7ZFQ0_PENO1         SAMWDSVENWDFQATEGAAV 593
TR|A0A1Q5UN98|A0A1Q5UN98_9EURO NAIWDSNESWDSQFTGAASG 673
TR|A0A0F7TSE6|A0A0F7TSE6_PENBI NTMLDSGESWESQFCGAAAG 660
                               .:: ** *.*: *   .*:
```
